# Neural-signature methods for structured EHR prediction

**DOI:** 10.1186/s12911-022-02055-6

**Published:** 2022-12-07

**Authors:** Andre Vauvelle, Paidi Creed, Spiros Denaxas

**Affiliations:** 1 Institute of Health Informatics, University College London, 222 Euston Road, London, UK; 2 BenevolentAI, 4-8 Maple St, London, UK

**Keywords:** Machine learning, Electronic healthcare records, Signature methods

## Abstract

Models that can effectively represent structured Electronic Healthcare Records (EHR) are central to an increasing range of applications in healthcare. Due to the sequential nature of health data, Recurrent Neural Networks have emerged as the dominant component within state-of-the-art architectures. The signature transform represents an alternative modelling paradigm for sequential data. This transform provides a non-learnt approach to creating a fixed vector representation of temporal features and has shown strong performances across an increasing number of domains, including medical data. However, the signature method has not yet been applied to structured EHR data. To this end, we follow recent work that enables the signature to be used as a differentiable layer within a neural architecture enabling application in high dimensional domains where calculation would have previously been intractable. Using a heart failure prediction task as an exemplar, we provide an empirical evaluation of different variations of the signature method and compare against state-of-the-art baselines. This first application of neural-signature methods in real-world healthcare data shows a competitive performance when compared to strong baselines and thus warrants further investigation within the health domain.

## Introduction

Prediction tasks defined on structured EHR data are a key focus for applications of Machine Learning in Healthcare, with the potential to improve patient outcomes through faster and more accurate diagnoses. Due to the rapidly increasing quantity and availability of EHR data, methods in deep learning are increasingly being utilised to model the complex interactions in a range of healthcare related predictive tasks. Due to the sequential nature of EHR data, RNNs have emerged as a key component in many recent state of the art methods. This paper introduces signature methods as a theoretically well-grounded method of extracting features from sequential structured EHR data. We provide an empirical evaluation of signature methods as a novel alternative to RNNs for disease prediction using data collected during routine healthcare encounters.

The signature transform maps a path (for example a time series) onto an infinite sequence of summary statistics. It is known that these terms completely characterise the path (up to translation) and that any function on the path can be modelled arbitrarily well by a linear function on the signature [21, 34]. In a machine learning context, this makes the signature a useful feature set with which to learn from. The signature has been successful across a range of predictive tasks involving time series data [37] in particular in the medical domain [38]. However, the signature method has not yet been applied to structure EHR data, most likely due to its high dimensionality posing computational challenges. To this end, we follow recent work that enables the signature to be used as a differentiable layer within a neural architecture enabling application in high dimensional domains where calculation would have previously been intractable [7].

In this paper we perform an empirical evaluation of signature methods as a novel alternative to RNNs for disease prediction using EHR data. We create a 90 day HF prediction task with data from the UK Biobank [9] to compare neural-signature models with various augmentations against RNN and bag of words baselines. The key results can be summarised as follows:

- Neural-signature methods are able to produce a competitive predictive performance when compared to RNN models, returning results over two separate corpora and metrics within one standard deviation
- Log-signature and lead-lag variants improve results from those similar to basic bag of words models to those comparable with RNNs
- Adding time-augmentations does not significantly effect model performance for both neural-signature and RNN models

## Related work

The methods previously used to address temporality in EHR can be roughly separated into three main areas;

*Discretization* This consists of splitting the continuous-time variables into discrete bins. Features are then calculated from the sub-sequences within each time period. For categorical data, the most common approach is to count the number of events.

*Neural approaches* Neural network approaches attempt to automatically learn a feature set that best describes the underlying data for a specific prediction task. [41] and [12] applied RNN variants to find results that reported improved performance over existing state-of-the-art methods.

RNN variants continue to play a role in more recent papers [1, 13, 14, 20, 35, 42, 47, 51]. Modifications include using bidirectional RNNs to reduce steps between dependencies, attention mechanisms to improve interpretability, facilitate combining with Convolutional Neural Network (CNN) models, and improved embeddings for visits with graph-based attention models. In all such papers, RNNs are used to handle the sequential aspect of structured EHR.

While it is clear that RNNs perform comparatively well in deep learning applications, an alternative set of methods is also worth discussing.

*Sequential feature extraction* This encompasses methods that are able to extract flat features from sequential data while retaining information relating to ordering. Despite being more popular in higher frequency data modalities such as streams data, previous works using structure EHR have explored; shapelets [52] and symbolic aggregate approximation [4] for adverse drug reaction prediction. More broadly, this includes methods such as the discrete Fourier transformation [43].

It is this category that signature methods can be considered to belong. A key advantage of signature methods is a strong theoretical groundwork showing the signatures usefulness in non-parametric hypothesis testing [11] and algebraic geometry [40]. Machine learning applications have also been demonstrated in a growing variety of domains [10] including: healthcare [3, 27, 28, 38], finance [24, 39], action recognition [32, 49] and hand-writing recognition [50].

## Data preprocessing and cohort

The UK Biobank [9] is a national population-based study comprising of 502,629 individuals. We extract a retrospective heart failure (HF) cohort using the same methodology as [19] which uses the previously-validated phenotyping algorithm in the CALIBER resource [18].

To form the sequential input data required for our predictive model, we extract primary and secondary diagnosis terms (ICD10), procedure terms (OPCS4) and, timestamps (“epistart”) from the UK Biobank inpatient dataset. Patient events are extracted with a buffer period of 90 days before HF diagnosis (for controls this is the HF diagnosis of its matched case) to exclude highly correlated events such as end of life care [29]. Events that occur at the same time are randomly ordered.

We create two separate corpora for each patient: PRIMDX is a corpus that only contains primary diagnosis terms, and PRIMDX-SECDX-PROC also includes secondary diagnoses and procedure terms. Since the number of events in each sequence is greater for the PRIMDX-SECDX-PROC cohort, this allows us to compare each of our methods’ ability to handle longer sequences with more complex and redundant information.

In Table 1 we provide a breakdown of the demographics of the matched cohort used in this study. In Appendix A we provide further details on the HF cohort extracted and the tokenization process of healthcare terms.

**Table 1:** A summary of the cohort used for HF prediction experiments

		HF	Controls	Total
# patients		5722	51,498	57,220
PRIMDX	# events	48,779	188,893	237,672
	Average # events per patient	8.52	3.67	4.15
	Unique tokens	–	–	2147
PRIMDX-SECDX-PROC	# events	184,036	616,066	800,102
	Average # events per patient	32.16	11.96	13.98
	Unique tokens	–	–	6235

## Methods

Let each patient record be denoted by the path ${\mathbf {x}} = (x^1_t, \ldots , x^d_t)$, where each value $x^i_t$ is real-valued and parameterised by $t \in [0,T]$.

Our objective is to classify each sequence with a binary variable which indicates whether the patient will develop heart failure within 90 days. The dimension of the path, *d*, is be determined by the maximum number of unique tokens as we represent each token with a one-hot-vector, such that only the dimension corresponding with the index of the vocabulary is one and with zeros everywhere else.

### Signature methods

The definition of the signature transform is as follows.

Let $T > 0$ and $0< t_1< t_2< \cdots< t_{n-1} < t_n = T$. Let $f_x = (f_x^1, \ldots , f_x^d): [0,T] \rightarrow {\mathbb {R}}^d$ be the unique continuous function such that $f_x(t_i)=x_i$ and is affine on the intervals between them. The signature is the infinite collection of iterated integrals

1 $$\begin{aligned}&\mathrm {Sig}({\mathbf {x}}) = \nonumber \\&\left( \left( \,\underset{0< t_1< \cdots< t_k < T}{\int \cdots \int } \prod _{j = 1}^k \frac{ d f^{i_j}}{ dt}(t_j) dt_j \right) _{1 \le i_1, \ldots , i_k \le d}\right) _{1 \le k}. \end{aligned}$$

The form of the signature in Eq. 1 can be broken down to help give the reader a better understanding as done in [10]. We can start by simplifying to a single index $i \in \{1, \ldots , d\}$. This reduces Eq. 1 to

2 $$\begin{aligned} \mathrm {Sig}({\mathbf {x}})^i = \int _{0<t<T}\frac{ d f^{i}}{dt}(t) dt. \end{aligned}$$

As this is a single integral and *f* is affine, the equation simply resolves to the increment of i-th coordinate of the path

3 $$\begin{aligned} \mathrm {Sig}({\mathbf {x}})^i = x^i_T - x^i_0. \end{aligned}$$

The double-interated integral considers any pair of coordinates $i,j \in \{1,\ldots , d\}$ such that

4 $$\begin{aligned} \mathrm {Sig}({\mathbf {x}})^{i,j}&= \int _{0<t<T}\mathrm {Sig}(\mathbf{x})^i\frac{ d f^{i}}{dt}(t) dt\end{aligned}$$

5 $$\begin{aligned}&= \int _{0<t_1<t_2<T}\frac{ d f^{i}}{ dt_1}(t_1) dt_1 \frac{d f^{j}}{dt_2}(t_2) dt_2 \end{aligned}$$

where we have used Eq. 2 and replaced *t* to denote the integration limits as

6 $$\begin{aligned} a< t_1< t_2< T = {\left\{ \begin{array}{ll} 0< t_1< t_2 \\ 0< t_2 < T \end{array}\right. } \end{aligned}$$

Notice that the integration limits in Eq. 6 correspond to the integration over a triangle. Going further this process can continue recursively and be interpreted as integrating over an increasingly high dimensional simplex. This real number is known as the k-fold iterated integral seen in Eq. 1

7 $$\begin{aligned} \mathrm {Sig}({\mathbf {x}})^{i_i,\ldots ,i_k}&= \int _{0<t<T}\mathrm {Sig}({\mathbf {x}})^{i_1,\ldots ,i_{k-1}}\frac{ d f^{i_k}}{dt}(t) dt \end{aligned}$$

8 $$\begin{aligned}&= \,\underset{0< t_1< \cdots< t_k < T}{\int \cdots \int } \prod _{j = 1}^k \frac{ d f^{i_j}}{ dt}(t_j) dt_j \end{aligned}$$

where the superscripts are members of the set

9 $$\begin{aligned} 1,\ldots ,k\in \{1,\ldots ,d\}. \end{aligned}$$

We can further simplify this form and remove the need for the integral when we consider the path as a series of linear segments in a piecewise linear path. For a single segment the signature can be expressed by the product of its increment

10 $$\begin{aligned} \mathrm {Sig}({\mathbf {x}})^{i_1,\ldots ,i_k}_{t,t+1} = \frac{1}{k!} \prod _{j = 1}^k (x^{i_j}_{t+1} - x^{i_j}_{t}). \end{aligned}$$

To calculate each signature term of the full path, we can use Chen’s Identity, which states that the signature of the entire path can be calculated from the signatures of its segments [30]

11 $$\begin{aligned} \mathrm {Sig}({\mathbf {x}})^{i_1,\ldots ,i_k}_{0,T} = \sum ^k_{j=0}\mathrm {Sig}(\mathbf{x})^{i_1,\ldots ,i_j}_{0,t}\mathrm {Sig}(\mathbf{x})^{i_1,\ldots ,i_k}_{t,T}. \end{aligned}$$

Using the signature as an infinite series in a machine learning pipeline would not be tractable. Instead, it is common to truncate the series to the k-th level, this is also known as the depth of the signature. This results in the finite collection of terms $\mathrm {Sig}({\mathbf {x}})^{i_1,\ldots ,i_k}$ where the multi-index is restricted to length *N*. For example a signature of depth 1 is the collection of *d* real numbers $\mathrm {Sig}({\mathbf {x}})^1, \ldots , \mathrm {Sig}({\mathbf {x}})^d$ and a signature of depth 2 is the collection of $d+d^2$ real numbers $\mathrm {Sig}({\mathbf {x}})^1, \ldots , \mathrm {Sig}({\mathbf {x}})^d, \mathrm {Sig}^{1,1}, \ldots , \mathrm {Sig}({\mathbf {x}})^{d,d}$.

The number of terms $\tau$, for any truncated signature of order *N* of a d-dimensional path, where $d \ge 1$, is the geometric series:

12 $$\begin{aligned} \tau = \sum _{k=0}^N d^k = \frac{d^{N+1}-1}{d-1} \end{aligned}$$

For structured EHR data with hundreds or thousands of unique terms, this poses a significant computational issue. In the next section, we highlight a number of variations that can be used to encourage information into lower order signature terms.

In Appendix B, we provide a further breakdown of the definitions provided here and explore an example in toy data to show how the signature terms describe sequential data. Theoretically, the signature terms are proven to uniquely describe any path up to translations (Proposition 1) and act as a universal non-linearity (Proposition 2). This latter property is shared with neural networks and allows us to reduce potentially complicated non-linear relationships between variables into linear ones.

### Signature variations

There is a body of variations on the standard signature transform that have been developed. Each can tailor the properties of the signature to be more suited for a certain task. [37] provides an overview of possible variations of the signature together with an empirical evaluation on streams data. Given the substantially greater dimensionality of structured EHR data, we restrict our investigation to the augmentations in Table 3 and the log-signature (Table 2).

#### Augmentations

An augmentation considers transforming our sequence of patient events ${\mathbf {x}} \in {\mathbb {R}}^d$ into one or several new sequences, *p*, whilst potentially changing the dimensionality of each path to *a*. In general, this can be described by the map

13 $$\begin{aligned} \phi : {\mathcal {S}}({\mathbb {R}}^{d})\rightarrow {\mathcal {S}}({\mathbb {R}}^a)^p. \end{aligned}$$

**Table 2:** Summary of augmentations

Augmentation	*a*	*p*	Length
Time	$d+1$	1	*n*
Basepoint	*d*	1	$n + 1$
Lead-lag	2*d*	1	2*n*
Learnt projections	*a*	*p*	*n*

The time augmentation consists of the concatenation of an extra dimension. As shown in Proposition 1, this can be used in the absence of any actual timestamps by simply using the index of the event in the sequence. In both cases, this removes the property of time-parameterisation invariance of the signature [31]. We also investigate applying actual time differences from prediction date to account for the irregularly sampled nature of the data. We follow [1] by applying the parameterised scaling function, $f(\Delta T)=T_{scale} log(\Delta T)$ capped a maximum $T_{max}$. $T_{scale}$ and $T_{max}$ control extreme time-deltas and are optimised as hyperparameters.

The basepoint [25] is used to remove the property of translational invariance. This property means that the signature of two paths separated by a constant translation will be the same. The basepoint also has a significant advantage for our pipeline as $\sim 20\%$ of pathways in the dataset used in this study have only a single event. Basepoint introduces an origin point at the start of each path and thus ensures each path has at least two points which is a requirement for calculating the signature.

The lead-lag augmentation [10, 22] adds shifted copies of the path as new coordinates. This augmentation explicitly captures the quadratic variation of the underlying process, an important concept for our data where the co-variance between medical concepts is known to be highly important to the underlying pathology of disease [16, 42]. A lag of a single timestep is described by the following augmentation

14 $$\begin{aligned} \phi ({\mathbf {x}}) = ((x_1,x_1),(x_2,x_1),(x_2,x_2), \dots ,(x_t,x_t)) \in {\mathcal {S}}({\mathbb {R}}^{2d}). \end{aligned}$$

The learnt projection can be described by the affine transformation or embedding, $\theta _A \in {\mathbb {R}}^{a \times d}$, such that

15 $$\begin{aligned} \phi ({\mathbf {x}}) = (\theta _A x_1, \theta _A x_2, \ldots , \theta _A x_n) \in {\mathcal {S}}({\mathbb {R}}^{a}). \end{aligned}$$

This reduces the dimensionality of the path to make the calculation of the signature tractable.

#### The log-signature

The log-signature corresponds with taking the formal logarithm of the signature in the algebra of formal power series [10]. Both the signature and its logarithm uniquely define a path (Proposition 1) but the log-signature does not hold the same universality property (Proposition 2) [32]. The log-signature maps to a smaller number of terms at each truncation level determined by Witt’s formula, which is shown in Appendix B.3.2 along with an example.

#### Deep signatures

For the affine transformations discussed in Equation 15, we briefly described a learning process. As detailed in recent works from [7], it is possible to train the affine transformations together with the signature transform through an end-to-end neural network architecture. Here, the signature acts as a non-parametric pooling function able to extract provably useful information from sequential data.

It is possible to calculate the gradient needed in this method as the signature can be formulated as a calculation tree of differentiable operations [25, 45].

The generalised function of the neural-signature model used in this work can be written as

16 $$\begin{aligned} f({\mathbf {x}};\Theta ) = \sigma \left( (g^{\theta _{fc}} \circ \mathrm {Sig}^N \circ \phi ^{\theta _A})({\mathbf {x}}) \right) \end{aligned}$$

where we have denoted that the learnt parameters as the weights of a fully connected neural network classifier $\theta _{fc}$ and elements of the affine transformation augmentation $\theta _A$. The sigmoid function is used to map the output activation to a [0,1] score.

## Experiments

As baselines, we consider a bag of words model with logistic regression as a commonly used most basic model, along with a GRU model, which is comparable with the state of the art *RETAIN* [14, 48]. We also include a GRU variation that incorporates the time delta augmentation.

Additionally, we consider the following signature models: the standard signature (S) provides the baseline for further variations, the log-signature (LS) removes the universality property (the fully connected neural network classifier still guarantees this overall) but greatly reduces the number of signature terms, the lead-lag (LL) augmentation encourages information about the quadratic variation into lower-order signature terms, the add time index augmentation (ATI) provides sensitivity to parameterization, the time delta (ATD) version goes further to account for non-uniform sampling rates. We limited the exploration on augmentations to the above after initial testing on validation data found the leag-lag augmentation to be most influential.

**Fig. 1:**
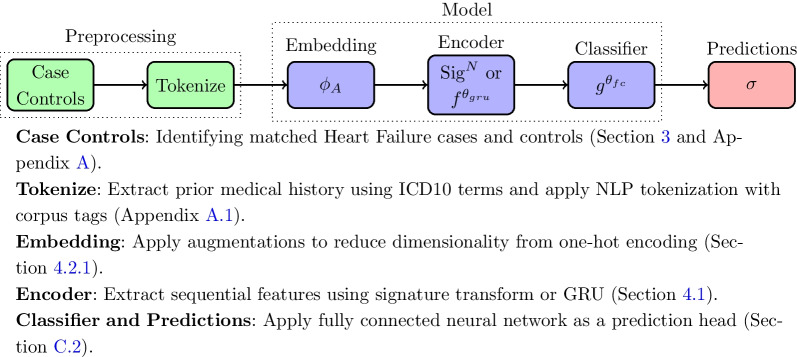
Overall experiment pipeline

**Table 3:** Sequential models compared

Variants	Description
$S^2$	Standard Signature with depth 2
$LS^2$	Log-signature—a condensed form of the signature
$LS^2 + LL$	*LS* with lead-lag augmentation—extracts quadratic variation
$LS^2 + LL + ATI$	$LS + LL$ with time index—removes time reparameterisation invariance
$LS^2 + LL + ATD$	$LS + LL$ with time delta—accounts for non-uniform sampling rate
$LS^3 + LL + ATD$	Increased truncation depth—more complex, sequential features
BoW OH LR	Most basic model with one-hot bagging and logistic regression
GRU	Baseline gated recurrent unit sequential model
GRU + ATD	Takes into account time differences

We use two metrics for evaluation; area under the receiver operator curve (AUROC) and area under the precision-recall curve (AUPRC).

Previous studies have shown that the AUROC can provide misleading results when there is considerable data imbalance, mainly if the number of negative examples is high, and we have a preference for identifying true positive examples [46]. This issue exists in our task due to the 1:9 case-control split and the increased benefit of correctly identifying HF cases over correctly identifying controls. The result of this class imbalance can cause AUROC to become inflated due to a high number of true negative cases. AUPRC is an alternative metric that captures the trade-off between precision and recall. Crucially, it ignores the number of true negatives allowing changes in performance to be seen without being diluted as in AUROC.

The signature variations explored are summarised together with the baselines in Table 3. Common to each model is the architecture shown in Fig. 1. Further details on implementation, including initialisation, activation functions, optimisation, hyperparameters, regularisation, and other such related details are found in Appendix C.

## Results

**Table 4:** Best performing models maximised for AUROC during hyperparameter optimisation

	PRIMDX			PRIMDX-SECDX-PROC		
Model	AUROC	AUPRC	# Params	AUROC	AUPRC	# Params
$S^2$	0.682 ± 0.011	0.211 ± 0.015	525,296	0.695± 0.005	0.247 ± 0.003	258,098
$LS^2$	0.687 ± 0.005	0.248 ± 0.014	637,906	0.704 ± 0.011	0.245 ± 0.019	167,101
$LS^2 + LL$	0.703 ± 0.007	0.241 ± 0.005	216,404	0.728 ± 0.011	0.300 ± 0.014	592,528
$LS^2 + LL + ATI$	0.700 ± 0.009	0.249 ± 0.018	864,817	0.724 ± 0.007	0.303 ± 0.009	545,474
$LS^2 + LL + ATD$	0.695 ± 0.013	0.254 ± 0.014	596,364	0.730 ± 0.008	0.304 ± 0.009	1,504,205
$LS^3 + LL + ATD$	0.698 ± 0.007	0.244 ± 0.017	7,015,643	**0.734** ± 0.008	0.300 ± 0.009	4,037,181
BoW OH LR	0.699 ± 0.007	0.252 ± 0.006	–	0.690 ± 0.015	0.300 ± 0.015	–
GRU	0.701 ± 0.009	0.241 ± 0.007	795,390	0.731 ± 0.008	0.293 ± 0.017	415,892
GRU + ATD	**0.710** ± 0.010	**0.275** ± 0.012	668,997	0.733 ± 0.008	**0.309** ± 0.012	489,151

Highest performing models in bold

Performance metrics evaluated on the test dataset with $\pm 1$ standard deviation calculated from cross validation scores

From Table 4, we observe similar predictive performance across signature models using lead-lag augmentations and GRU models over all corpora, with all metrics from the two sets of models remaining within one standard deviation of performance seen on the validation data. All models perform the same or better on the larger PRIMDX-SECDX-PROC cohort, but more complex models gain a more significant benefit from the added data.

The addition of time augmentations does not show a consistent improvement in performance over just applying the lead-lag augmentation, and there is no consistent difference between adding a time index and time delta. Increasing the depth of the signature to three also shows no significant increase in performance. Signature models perform similar to the bag of words baselines without the lead-lag transform.

### Data ablation study

Our final set of experiments evaluates how the models perform as the volume of data is reduced. For the data ablation study, each trial randomly samples a proportion of the training and validation dataset. For each proportion a new set of hyperparameters is found for each model.

The model parameters and hyperparameters are trained using 5-fold cross validation on the sub-sample while the remaining data is unused. The ablation study test data remains the same as the main experiment.

Again, results are broadly similar for both models except for three points where the two models produce performance outside of one standard deviation of validation performance. Notably, at 20% data ablation for AUPRC, the signature model has a 21.0% higher score with 0.283 versus 0.237 for the GRU. Overall, both models’ performance begins to saturate at $\sim 20\%$ for both metrics, and the results show no conclusive trend as to which model performs best as the amount of training data is reduced.

## Discussion

Given the properties that the two methods share these results might not come as a surprise. However, without the lead-lag augmentation the performance of the signature models drop significantly. This confirms the prior belief that the quadratic variance of the path plays an important role in structured EHR HF prediction. This could correspond with encouraging features that describe changing comorbidities to be present in the lower order terms of the signature.

For the data ablation study, we expected the signature model to outperform the GRU baseline however, results for both methods are similar. Our prior hypothesis was partly motivated by the success of signature methods in previous shallow machine learning tasks [38]. A key difference in our task could be the high dimensionality and reliance on embeddings to make the signature tractable. The need to train these embeddings is likely data-intensive but could benefit from initialisation using pretrained *word*2*vec* embeddings as has been shown for RNNs [12].

### Comparison to previous literature

Comparing the results in this paper directly to previous work is challenging due to the use of different underlying study designs, populations, and incomplete definitions of cohorts and outcomes [17]. We note that previous works investigating sequential models for predicting HF on structured EHR data have found greater performance [14, 44, 48]. In particular, these works also show a more significant performance difference between bag of words baselines and RNN based architectures. Again, differences in the features and data sources used make comparisons difficult. For example, we could compare against Solares et al. [48], which also uses data from a multi-center UK EHR data source and achieves 0.951 AUROC using the RNN based model RETAIN. However, we must consider that the authors also include primary care and demographics data, which could influence prediction performance independent of model choice. The large US multi-center study by [44] show RETAIN achieving a more comparable AUROC of 0.769 on US healthcare care with a balanced cohort with 14,500 cases and with only diagnosis codes provided for as prediction input. The same model achieved an AUROC of 0.822 when trained and tested on the full cohort of 152,790 cases and 1,152,517 controls with diagnoses, demographic, medication, and surgery data.

In this work, we have restricted our work to prediction on high dimensional structured EHR data. Signature methods have shown success in related health prediction applications but with lower dimensional, high frequency data domains including: mood ratings for Bipolar and Borderline Personality Disorder [3], brain imaging data for Alzheimer’s disease [36] and physiological data for Sepsis prediction [38]. Future work could look to expand signature method applications within similar domains such as ECG signals diagnosis [6] and prediction systems for biogas production [8].

## Conclusion

Given the prevalence of RNNs in current structured EHR architectures, any improvement in this fundamental component is likely to influence future work significantly. A substantial body of theory motivates the use of signature transforms to represent sequential data, and previous works have shown them to have strong empirical performance. In particular, recent works on neural-signature architectures have enabled their applications on high-dimensional data.

This work is the first to show that neural-signature methods with dimensional reduction before the transform are competitive on high dimensional structured EHR data. Using an HF prediction task, we evaluated the signature transforms as an alternative to RNNs that provide a predictive and compact representation of sequential structured EHR data. We show that the signature achieves comparable performance to RNNs and that the performance of both models saturates with a similar number of training examples. While the signature originates from perhaps abstract theory, empirically, it can successfully compete with the current state-of-the-art architectures.

## Figures and Tables

**Fig. 2:**
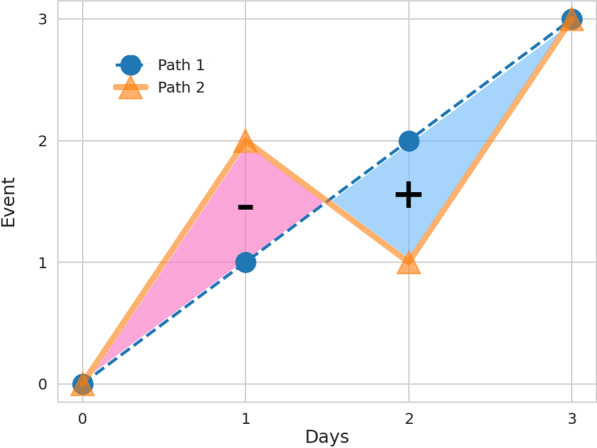
A plot of two event paths with path 1 also being the chord that joins the two end points of path 2. The Levy area is negative above the chord and positive above

## Data Availability

The data that support the findings of this study are available from the UK Bioank (https://www.ukbiobank.ac.uk/) but restrictions apply to the availability of these data, which were used under license for the current study, and so are not publicly available. For re-using these data, an application must be made directly to the UK Biobank. Code is available at https://github.com/andre-vauvelle/doctor-signature.

## Appendix A: Cohort

The HF cohort excludes patients with: self-reported prevalent HF cases, those who died during the study period, heart failure diagnoses outside the study period between 1997 and 2015. The cases are matched to controls on assessment center, year of recruitment, sex, and year of birth. Controls use an index date that is assigned to the date of HF diagnosis for its matched case. We randomly sampled the total potential control population of 496,892 with the above matching criteria to create a cohort with a ratio of 1:9 cases to controls. This resulted in 5722 cases and 51,498 controls, where 15 cases could not be matched.

### A.1 Tokenization

We follow a similar practice to NLP tokenization, using tags to identify whether a code comes from a particular corpus. This allows us to distinguish between primary and secondary codes with the same ICD10 term and introduce additional tokens to indicate if there is no data present. The tokenization process also applies two measures to reduce the vocabulary size; limit the length of all terms to 4 characters and a minimum count of 5. This is possible because many ICD10 and OPCS contain extension codes or additional sub-chapter codes which are not commonly used across healthcare providers and often contain details that indicate minor differences between terms. The tokenizer vocabulary is fitted on the training data, and any subsequent code that is not present in the vocabulary is assigned an out-of-vocabulary token in place.

## Appendix B: Signature methods

### B.1 A geometric intuition and exploration in toy data

Previously in Eq. 3 we showed that the first level resolves to the increment of a path in a single dimension. It is also possible to find a geometric interpretation for 2nd level terms in Eq. 2. For 2nd level terms where $i=j$ we find $\mathrm {Sig}({\mathbf {x}})^{i,j} = (x^i_T - x^i_0)^2/2$ which is simply the area of a triangle sided with the increment in the coordinate. If $i\ne j$ then $\mathrm {Sig}({\mathbf {x}})^{i,j} = (x^i_T - x^j_0)^2/2$. Another geometric comparison can be made here by considering the Lévy area, which is the signed area shown in Fig. 2. This area illustrates the 2nd order terms through the following equation

17 $$\begin{aligned} A_{levy} = \frac{1}{2}(\mathrm {Sig}({\mathbf {x}})^{1,2}. - \mathrm {Sig}({\mathbf {x}})^{2,1}). \end{aligned}$$

The Fig. 2 can be related to our task if we consider the line (path 2) and its cord (path 1) to be two separate patient pathways with three unique events (1,2,3), but for path 2 the order of events 1 and 2 are swapped. When classifying a patient pathway with an HF prediction, the order of events may hold vital information. However, when we calculate the terms of the signature in Table 5, we see that the two paths are only separable when taking a signature of depth 3.

Taking the geometric interpretation to higher orders becomes more complicated, but for additional interpretable relations, we refer the reader to [10] which expands on the ideas here to include relations to statistic moments. It is clear that these signature terms are extracting information about the order and that higher-order signature terms can be used to discriminate different paths better. It is also possible to observe how the terms of the signature change by drawing a path using an online tool^1^.

**Table 5:** Signature terms up the 3rd level for the paths in Fig. 2

Sig Terms	Path 1	Path 2	Different?
(1,)	3	3	FALSE
(2,)	3	3	FALSE
(1, 1)	4.5	4.5	FALSE
(1, 2)	4.5	4.5	FALSE
(2, 1)	4.5	4.5	FALSE
(2,2)	4.5	4.5	FALSE
(1, 1, 1)	4.5	4.5	FALSE
(1, 1, 2)	4.5	4	TRUE
(1, 2, 1)	4.5	5.5	TRUE
(1, 2, 2)	4.5	5.5	TRUE
(2, 1, 1)	4.5	4	TRUE
(2, 1, 2)	4.5	2.5	TRUE
(2, 2, 1)	4.5	5.5	TRUE
(2, 2, 2)	4.5	4.5	FALSE

### B.2 Key properties and caveats for signature methods

A natural extension to our toy example would be to ask if the signature can be used to discriminate between any path. In fact, it has been shown that a path is essentially defined by its signature and that almost no information is lost. There are two key properties of the signature that make its use interesting for path-like data.

#### Proposition 1

(Uniqueness of signature [23]). Given ${\mathbf {x}} = (x_1, \ldots , x_n)$ then the map ${\mathbf {x}} \rightarrow \mathrm {Sig}^{\infty }((1,x_1),(2,x_2),\ldots ,(x,x_n))$ uniquely determines ${\mathbf {x}}$ up to translations.

For the purpose of this statement we have introduced the two concepts of time-augmenting a path and of translational invariance. Both of which will be expanded on in Section B.3.

#### Proposition 2

(Universal non-linearity [39]) Let *F* be a real-valued continuous function on continuous piecewise smooth paths in ${\mathbb {R}}^d$ and ${\mathcal {K}}$ be a compact set of such paths. Furthermore assume that $x_0 = 0$ for all ${\mathbf {x}} \in {\mathcal {K}}$. (To remove the translational invariance.) Let $\epsilon > 0$. Then there exists a linear functional *L* such that for all ${\mathbf {x}} \in {\mathcal {K}}$

18 $$\begin{aligned} |F({\mathbf {x}})-L(\mathrm {Sig}({\mathbf {x}})| < \epsilon . \end{aligned}$$

In other words, we know that we can find a linear function that when applied to the signature of a path can approximate any function applied to that same path. This powerful property is shared with neural network approaches and allows us to reduce potentially complicated non-linear relationships between variables into the linear ones.

We refer the interested reader to Appendix A of [7] for an expanded summary.

### B.3 Variations on the signature transform

There is a growing body of variations on the standard signature transform that have been developed. Each can tailor the properties of the signature to be more suited for a certain task. [37] gives an overview of the variations on the signature transform and proposes a generalised framework for extracting signature features for time-series data.

19 $$\begin{aligned} {\mathbf {y}}_{i,j,k} = (\rho _{post} \circ \mathrm {Sig}^N \circ \rho _{pre} \circ W^{i,j} \circ \phi ^k)({\mathbf {x}}). \end{aligned}$$

Here we have introduced a number of new terms. Each of which will be explained in more detail and discussed in relation to our data. Briefly; $\phi$ is known as an augmentation, *W* describes a windowing operation, $\rho$ describes a re-scaling operation both pre and post calculating the signature. Finally, $\mathrm {Sig}^N$ represents either the now familiar signature operation up to depth N or the log-signature variation, which will be introduced later in this section. The indices (*i*, *j*) refer to the signature terms of windowed operations, with ${\mathbf {y}}_{i,j,k}$ reducing to the more familiar signature terms up to level k, ${\mathbf {y}}_k$, when a global window is used. For our work, we will focus on only two aspects of this generalised framework, augmentations and the log-signature, leaving the exploration of the additional steps to future work.

#### B.3.1 Augmentation

An augmentation considers transforming our sequence of patient events ${\mathbf {x}} \in {\mathbb {R}}^d$ into one or several new sequences, *p*, whilst potentially changing each paths dimensionality to *a*. In general this can be described by the map

20 $$\begin{aligned} \phi : {\mathcal {S}}({\mathbb {R}}^{d})\rightarrow {\mathcal {S}}({\mathbb {R}}^a)^p. \end{aligned}$$

There are a number of different augmentations that have been used for continuous time series data.

The augmentations in Table 2 are broadly separated in two categories. Fixed augmentations consider using a transformation that does not vary after initialisation and learnt augmentations which include learnable parameter weights that are trained as part of the model fitting process. We start by explaining the two previously mentioned augmentations, time and basepoint augmentations.

The time augmentation is the addition of an extra dimension that is dependant on the order of the sequence. As shown in Proposition 1, this can be used in the absence of any actual timestamps by simply using the index of the event in the sequence

21 $$\begin{aligned} \phi ({\mathbf {x}})= \big ((1,x_1),\dots ,(n,x_n) \big ) \in {\mathcal {S}}({\mathbb {R}}^{d+1}) \end{aligned}$$

or if timestamps are available

22 $$\begin{aligned} \phi _{t}({\mathbf {x}})= \big ((t_1,x_1),\dots ,(t_n,x_n) \big ) \in {\mathcal {S}}({\mathbb {R}}^{d+1}). \end{aligned}$$

In both cases this removes the property of time-parameterisation invariance of the signature [31]. Meaning that without time augmentation the signature only encodes the order in which events arrive and does not consider the when event arrives. This could potentially be a significant factor for our task. For example, consider two patient records of the same sequence of HF related inpatient admissions, if in one sequence the frequency of these admission was much higher it might suggest a increased disease progression. Using the actual timestamps allows the signature to account for the irregularly sampled nature of the data.

The basepoint [25] and invisibility augmentations [49] are both created with the goal of removing the property of translational invariance. This says that the signature of two paths separated by a constant translation will be the same. In [37] a comparison shows that the invisibility augmentation has essentially the same performance as the basepoint but increases the dimensionality of the path, which is of concern since the signature scales with ${\mathcal {O}}(d^N)$ as seen in Eq. 12. The basepoint also has a significant advantage for our pipeline as $\sim 20\%$ of pathways in the dataset used in this study have only a single event. Basepoint introduces an origin point at the start of each path and thus ensures each path has at least two points which is a requirement for calculating the signature.

23 $$\begin{aligned} \phi _{t}({\mathbf {x}})= (0, x_1,\dots , x_n ) \in {\mathcal {S}}({\mathbb {R}}^{d}). \end{aligned}$$

The lead-lag augmentation [10, 22] adds shifted copies of the path as new coordinates. This explicitly captures the quadratic variation of the underlying process, an important concept for our data where the co-variance between medical concepts or otherwise described as comorbidities, are known be highly important to the underlying pathology of disease [16, 42]. A lag of a single timestep is described by the following augmentation

24 $$\begin{aligned} \phi ({\mathbf {x}})= ((x_1,x_1),(x_2,x_1),(x_2,x_2),\dots ,(x_n,x_n)) \in {\mathcal {S}}({\mathbb {R}}^{2d}). \end{aligned}$$

Lead-lag comes at a high cost as the dimensionality and length of the path is doubled. The remaining augmentations provide a potential remedy for this as they propose reducing the dimensionality the path before calculating the signature. We only consider stream-preserving neural networks that take one projection, *p*, as the dimensionality of our paths is orders of magnitude higher than that considered in [37].

stream-preserving neural networks have also been used as an initial stage in previous literature on structured EHR data HF prediction tasks and provide a strong basis for comparison [15, 19, 48]. In its most basic form, a stream preserving neural network can be created by introducing a learnable affine transformation, $\theta _A \in {\mathbb {R}}^{a \times d}$, such that

#### B.3.2 The log-signature

The log-signature is a compressed version of the signature that only retains the Lyndon words of the index series [2]. As an example, we show the terms of the log signature up to depth 2 as

25 $$\begin{aligned} \begin{aligned} \mathrm {log}~\mathrm {Sig}^2({\mathbf {x}})&= \left( 1, \mathrm {Sig}(\mathbf{x})^1, \ldots , \mathrm {Sig}({\mathbf {x}})^d,\right. \\ {}&\quad \frac{1}{2}\left( \mathrm {Sig}({\mathbf {x}})^{1,2}- \mathrm {Sig}(\mathbf{x})^{2,1}\right) , \ldots , \\&\quad \frac{1}{2}\left( \mathrm {Sig}({\mathbf {x}})^{1,d}- \mathrm {Sig}({\mathbf {x}})^{d,1}\right) , \dots , \\&\quad \left. \frac{1}{2}\left( \mathrm {Sig}({\mathbf {x}})^{d-1,d}- \mathrm {Sig}({\mathbf {x}})^{d,d-1}\right) \right) \end{aligned} \end{aligned}$$

Here we can notice that the 2nd order terms of the log signature directly correspond with the Lévy area in Equation 17.

The number of terms in this more compact form of the signature can be generalised with the following.

##### Proposition 3

The number of terms of the truncated log-signature $\tau _{log}$, of order N of a d-dimensional path, where $d \ge 1$ is given by Witt’s formula [33]

26 $$\begin{aligned} \tau _{log} = w(d,N)=\sum _{k=1}^{N}\frac{1}{k}\sum _{i|k}\mu \left( \frac{k}{i}\right) d^i \end{aligned}$$

where $\mu$ is the Möbus function.

This results in almost a third of the output signature terms when calculating the signature for a path with $d=50$ at depth 3 ($\tau _{log}=42,925$ vs. $\tau = 127,550$).

## Appendix C: Experiments

### C.1 Augmentations

After tokenization, we project the sequence into a lower-dimensional representation with an embedding, which is the learnable affine transformation we describe in Equation 15. For neural-signature and GRU baselines, this is initialized with a Xavier distribution and trained in an end-to-end fashion and updated during gradient descent.

We experiment with applying different combinations of previously discussed augmentations: lead-lag, index, or time delta parameterization. If both lead-lag and time parameterization are applied, then time parameterization is always done first, such that $\phi (x) \in {\mathbb {R}}^{2(a+1)}$. This sequence of embedded codes is used as input to the encoder stage.

### C.2 Classifier

Dropout is applied to the outputs of fully connected hidden layers and determined by hyperparameter optimization.

The bag of words models use a logistic regression classifier with the sklearn implementation of L-BFGS-B and L2 regularisation.

### C.3 Implementation

The experiments were implemented using: PyTorch and Signatory [25]. Models were trained on various GPU cards available through the UCL Department of Computer Science High Performance Computing Cluster, using distributed asynchronous hyperparameter optimization and MongoDB. Code used for the project, including full details on hyperparameter bounds are available at https://github.com/andre-vauvelle/doctor-signature.

### C.4 Training and validation schema

All models are trained by minimizing binary cross-entropy loss with Adam [26]. We also stop training after five epochs if validation loss does not improve. The best model is then taken for evaluation on the test dataset.

Nested cross validation is used to evaluate our models and baselines. The data is split into training, validation, and held-out test sets with a 90% training and validation, 10% test data split. Training and validation sets are used in an inner loop with 5-fold cross validation to train model hyperparameters. Each fold is stratified such that we preserve the percentage of samples for each class. We use a Bayesian hyperoptimization algorithm, Tree-structured Parzen Estimator, to reduce the number of evaluations needed. We fix the number of trails for each model at 100 [5]. We then select the hyperparameters with the highest average AUROC score across a 5-fold. Test data is unseen until the final evaluation, where we use it to make predictions using models trained with optimal hyperparameters from the inner validation loop on one fold of data.

## References

[1] Alvin R (2018). Scalable and accurate deep learning with electronic health records. npj Digit Med.

[2] Améndola C, Friz P, Sturmfels B. Varieties of signature tensors. Forum Math Sigma. 2019;7:e10. 10.1017/fms.2019.3. arXiv: 1804.08325.

[3] Arribas Perez I, Saunders K, Goodwin G, Lyons T (2018). A signature-based machine learning model for bipolar disorder and borderline personality disorder. Transl Psychiatry.

[4] Bagattini F, Karlsson I, Rebane J, Papapetrou P. A classification framework for exploiting sparse multi-variate temporal features with application to adverse drug event detection in medical records. BMC Med Inf Decis Ma, 2019. 10.1186/s12911-018-0717-4. https://www.ncbi.nlm.nih.gov/pmc/articles/PMC6327495/. 10.1186/s12911-018-0717-4 PMC6327495 30630486

[5] Bergstra JS, Bardenet R, Bengio Y, Balázs K. Algorithms for hyper-parameter optimization. In: Shawe-Taylor J, Zemel RS, Bartlett PL, Pereira F, Weinberger KQ, editors, Advances in neural information processing systems 24, p. 2546–54. Curran Associates, Inc., 2011. http://papers.nips.cc/paper/4443-algorithms-for-hyper-parameter-optimization.pdf.

[6] Beritelli F (2018). A novel training method to preserve generalization of RBPNN classifiers applied to ECG signals diagnosis. Neural Netw.

[7] Bonnier P, Kidger P, Arribas Perez I, Salvi C, Lyons T. Deep signature transforms, 2019. arXiv 1905.08494[cs, stat].

[8] Capizzi G, Sciuto GL, Napoli C, Woźniak M, Susi G (2020). A spiking neural network-based long-term prediction system for biogas production. Neural Netw.

[9] Cathie S (2015). UK Biobank: an open access resource for identifying the causes of a wide range of complex diseases of middle and old age. PLOS Med.

[10] Chevyrev I, Kormilitzin A. A primer on the signature method in machine learning, 2016. arXiv: 1603.03788 [cs, stat].

[11] Chevyrev I, Oberhauser H. Signature moments to characterize laws of stochastic processes, 2018. arXiv: 1810.10971 [math, stat].

[12] Choi E, Bahadori MT, Schuetz A, Stewart WF, Sun J. Doctor AI: predicting clinical events via recurrent neural networks. 2016. arXiv: 1511.05942 [cs]. PMC5341604 28286600

[13] Choi E, Bahadori MT, Song L, Stewart WF, Sun J. GRAM: graph-based attention model for healthcare representation learning. 2017. arXiv: 1611.07012 [cs, stat]. 10.1145/3097983.3098126 PMC7954122 33717639

[14] Choi E, Bahadori MT, Sun J, Kulas J, Schuetz A, Stewart W. RETAIN: an interpretable predictive model for healthcare using reverse time attention mechanism. In: Lee DD, Sugiyama M, Luxburg UV, Guyon I, Garnett R, editors, Advances in Neural Information Processing Systems 29, p. 3504–12. Curran Associates, Inc., 2016b. http://papers.nips.cc/paper/6321-retain-an-interpretable-predictive-model-for-healthcare-using-reverse-time-attention-mechanism.pdf.

[15] Choi E, Schuetz A, Stewart WF, Sun J. medical concept representation learning from electronic health records and its application on heart failure prediction. 2016c. arXiv: 1602.03686 [cs].

[16] Choi E, Xu Z, Li Y, Dusenberry MW, Flores G, Xue Y, Dai AM. Learning the graphical structure of electronic health records with graph convolutional transformer. 2020. arXiv: 1906.04716 [cs, stat].

[17] Colin W, George H (2014). The effects of data sources, cohort selection, and outcome definition on a predictive model of risk of thirty-day hospital readmissions. J Biomed Inf.

[18] Denaxas S, George J, Herrett E, Shah AD, Kalra D, Hingorani AD, Kivimäki M, Timmis AD, Smeeth L, Hemingway H (2012). Data resource profile: cardiovascular disease research using linked bespoke studies and electronic health records (CALIBER). Int J Epidemiol.

[19] Denaxas S, Stenetorp P, Riedel S, Pikoula M, Dobson R, Hemingway H. Application of clinical concept embeddings for heart failure prediction in UK EHR data. 2018. arXiv: 1811.11005 [cs, stat].

[20] Ester M, Pedreschi D (2018). Health-ATM: a deep architecture for multifaceted patient health record representation and risk prediction.

[21] Friz PK, Victoir NB. multidimensional stochastic processes as rough paths: theory and applications. In: Cambridge studies in advanced mathematics. Cambridge University Press, Cambridge, 2010. 10.1017/CBO9780511845079.

[22] Guy F, Ben H, Terry L (2016). Discretely sampled signals and the rough Hoff process. Stoch Process Appl.

[23] Hambly B, Lyons T (2010). Uniqueness for the signature of a path of bounded variation and the reduced path group. Ann Math.

[24] Kalsi J, Lyons T, Arribas Perez I. Optimal execution with rough path signatures. 2019. arXiv: 1905.00728 [q-fin].

[25] Kidger P, Lyons T. Signatory: differentiable computations of the signature and logsignature transforms, on both CPU and GPU. 2020. arXiv: 2001.00706 [cs, stat].

[26] Kingma DP, Ba J. Adam: a method for stochastic optimization. 2017. arXiv: 1412.6980 [cs].

[27] Kormilitzin AB, Saunders KEA, Harrison PJ, Geddes JR, Lyons TJ. Application of the signature method to pattern recognition in the CEQUEL clinical trial. 2016. arXiv: 1606.02074 [stat].

[28] Kormilitzin A, Saunders KEA., Harrison PJ, Geddes JR, Lyons T. Detecting early signs of depressive and manic episodes in patients with bipolar disorder using the signature-based model. 2017. arXiv: 1708.01206 [stat].

[29] Kourou K, Exarchos TP, Exarchos KP, Karamouzis MV, Fotiadis DI (2014). Machine learning applications in cancer prognosis and prediction. Comput Struct Biotechnol J.

[30] Kuo-Tsai C (1958). Integration of paths-a faithful representation of paths by noncommutative formal power series. Trans Am Math Soc.

[31] Levin D, Lyons T, Ni H. Learning from the past, predicting the statistics for the future, learning an evolving system. 2016. arXiv: 1309.0260 [q-fin].

[32] Liao S, Lyons T, Yang W, Ni H. Learning stochastic differential equations using RNN with log signature features. 2019. arXiv: 1908.08286 [cs, stat].

[33] Lothaire M, eds. Combinatorics on Words. Cambridge Mathematical Library. Cambridge University Press, Cambridge, 2nd ed. 1997. 10.1017/CBO9780511566097. https://www.cambridge.org/core/books/combinatorics-on-words/6FEBB4FCCB43895CCEFA8D69A0983374.

[34] Lyons TJ, Caruana MJ, Lévy T. Differential equations driven by rough paths: Ecole d’Eté de Probabilités de Saint-Flour XXXIV-2004. École d’Été de Probabilités de Saint-Flour. Springer-Verlag, Berlin Heidelberg, 2007. 10.1007/978-3-540-71285-5. https://www.springer.com/gp/book/9783540712848.

[35] Ma F, Chitta R, Zhou J, You Q, Sun T, Gao J. Dipole: diagnosis prediction in healthcare via attention-based bidirectional recurrent neural networks. In: Proceedings of the 23rd ACM SIGKDD international conference on knowledge discovery and data mining - KDD ’17, p. 1903–11, 2017. 10.1145/3097983.3098088. arXiv: 1706.05764.

[36] Moore PJ, Lyons TJ, Gallacher J (2019). For the Alzheimer’s disease neuroimaging initiative. Using path signatures to predict a diagnosis of Alzheimer’s disease. PLOS ONE.

[37] Morrill J, Fermanian A, Kidger P, Lyons T. A generalised signature method for time series. 2020. arXiv: 2006.00873 [cs, stat].

[38] Morrill J, Kormilitzin A, Nevado-Holgado A, Swaminathan S, Howison S, Lyons T. The signature-based model for early detection of sepsis from electronic health records in the intensive care unit. In: 2019 computing in cardiology conference. 2019. 10.22489/CinC.2019.014.

[39] Perez Arribas P. Derivatives pricing using signature payoffs., September 2018. arXiv: 1809.09466 [q-fin].

[40] Pfeffer M, Seigal A, Sturmfels B. Learning paths from signature tensors. 2018. arXiv: 1809.01588 [cs, math, stat].

[41] Pham T, Tran T, Phung D, Venkatesh S. DeepCare: a deep dynamic memory model for predictive medicine. In: James B, Latifur K, Takashi W, Gill D, Joshua ZH, Ruili W, editors. Advances in knowledge discovery and data mining, lecture notes in computer science. Cham: Springer International Publishing; 2016; p. 30–41. 10.1016/j.jbi.2017.04.001.

[42] Qian Z, Alaa AM, Bellot A, Rashbass J, van der Schaar M . Learning dynamic and personalized comorbidity networks from event data using deep diffusion processes. 2020. arXiv: 2001.02585 [cs, stat].

[43] Rakesh A, Christos F, Arun S, Lomet DB (1993). Efficient similarity search in sequence databases. Foundations of data organization and algorithms, lecture notes in computer science.

[44] Rasmy L, Wu Y, Wang N, Geng X, Zheng WJ, Wang F, Wu H, Xu H, Zhi D (2018). A study of generalizability of recurrent neural network-based predictive models for heart failure onset risk using a large and heterogeneous EHR data set. J Biomed Inf.

[45] Reizenstein JF. Iterated-integral signatures in machine learning. In: PhD thesis, University of Warwick, 2019.

[46] Saito T, Rehmsmeier M (2015). The precision-recall plot is more informative than the ROC plot when evaluating binary classifiers on imbalanced datasets. PLOS ONE.

[47] Shuai X, Junchi Y, Mehrdad F, Le S, Xiaokang Y, Hongyuan Z (2019). Learning time series associated event sequences with recurrent point process networks. IEEE Trans Neural Netw Learn Syst.

[48] Solares JRA, Raimondi FED, Zhu Y, Rahimian F, Canoy D, Tran J, Gomes ACP, Payberah AH, Zottoli M, Nazarzadeh M, Conrad N, Rahimi K, Salimi-Khorshidi G (2020). Deep learning for electronic health records: a comparative review of multiple deep neural architectures. J Biomed Inf.

[49] Yang W, Lyons T, Ni H, Schmid C, Jin L. Developing the path signature methodology and its application to landmark-based human action recognition. 2019. arXiv 1707.03993 [cs].

[50] Zecheng X, Zenghui S, Lianwen J, Hao N, Terry L (2018). Learning spatial-semantic context with fully convolutional recurrent network for online handwritten Chinese text recognition. IEEE Trans Pattern Anal Mach Intell.

[51] Zhang J, Kowsari K, Harrison JH, Lobo JM, Barnes LE (2018). Patient2Vec: a personalized interpretable deep representation of the longitudinal electronic health record. IEEE Access.

[52] Zhao J, Papapetrou P, Asker L, Boström H (2017). Learning from heterogeneous temporal data in electronic health records. J Biomed Inf..

